# Role of miR-128-3p and miR-195-5p as biomarkers of coronary artery disease in Indians: a pilot study

**DOI:** 10.1038/s41598-024-61077-4

**Published:** 2024-05-24

**Authors:** Raj Rajeshwar Choudhury, Harshi Gupta, Sudha Bhushan, Archna Singh, Ambuj Roy, Neeru Saini

**Affiliations:** 1 Functional Genomics Unit, CSIR-Institute of Genomics and Integrative Biology (IGIB), Mall Road, 110007 Delhi, India; 2 Academy of Scientific & Innovative Research (AcSIR), Ghaziabad, 201002 India; 3 Department of Biochemistry, All India Institute of Medical Sciences, Ansari Nagar, New Delhi, 110029 India; 4 Department of Cardiology, All India Institute of Medical Sciences, Ansari Nagar, New Delhi, 110029 India

**Keywords:** Coronary artery disease, Atherosclerosis, MicroRNA, miR-128-3p, miR-195-5p, Biomarker, Biomarkers, Cardiology

## Abstract

Coronary artery disease (CAD) imposes a significant economic burden in developing countries like India. Timely diagnosis and treatment should be prioritized to mitigate the disease. Current diagnostic tools being invasive and less specific raise the need to develop less invasive and more reliable molecular biomarkers. MicroRNAs (miRNAs) are an emerging class of molecules that can serve as a potential source of non-invasive biomarkers for CAD. The objective of this study was to determine the potential of circulatory miRNAs as diagnostic biomarkers in CAD. In this study, we have reported two microRNAs, miR-128-3p and miR-195-5p in the serum of CAD patients in Indian Population. A total of 124 subjects were recruited which included 89 angiographically proven CAD patients and 35 control subjects. Our results show a significant decrease in the levels of miR-128-3p in CAD patients while there were no significant changes in the levels of miR-195-5p. Further bioinformatics analysis revealed the potential role of miR-128-3p in cholesterol homeostasis. Altered homeostasis due to cholesterol accumulation in macrophages is the driving force behind formation of foam cells which in turn accelerates the progression of CAD. Here, we have shown that miR-128-3p increases cholesterol levels in macrophages by decreasing cholesterol efflux in-vitro.

## Introduction

Coronary artery disease is characterized by obstruction of coronary arteries due to gradual buildup of atherosclerotic plaque in them^1^. Plaque can lead to stenosis that limits the blood flow causing ischemia. It can also provoke thrombus formation leading to interruption in blood flow and myocardial infarction. CAD is a leading cause of mortality in low and middle-income countries like India (death rate of 145 per 100,000 individuals) and poses a big economic burden^2^. Most of the CAD related deaths occur due to lack of timely diagnosis and medical intervention^2–4^.

Currently several diagnostic approaches are being used for the detection of CAD such as electrocardiogram (ECG), echocardiogram, angiography and other molecular biomarkers such as cardiac troponin (cTnT), Creatine kinase-mB (CK-MB), C-reactive protein (CRP)^5^. However, most of the diagnostic methods are invasive or have limitations based on their specificity, sensitivity or their half-life^5^. Therefore, there is an urgent need for some reliable, non-invasive biomarkers for optimal detection and management of CAD.

MicroRNAs (miRNA) are a class of small noncoding RNAs (18 to 25 nucleotides) that mediate gene silencing by binding to 3' Untranslated region (UTR) of target genes^6^. They are involved in diverse biological processes including developmental timing, differentiation, proliferation, cell death and metabolism^6^. Circulatory micro RNAs are remarkably stable in the serum and are being explored as useful biomarkers for various human disease^7,8^. This stability for extended periods of time is achieved due to two mechanisms, i.e., formation of a ribonucleoprotein complex with Argonaute proteins and incorporation of miRNAs into exosomes where they are protected against degradation by RNAses in the blood^9^. Circulating miRNAs are also specific to tissue and disease type which further increase their potential as a diagnostic marker^10,11^. Recently few studies have also described the role of circulating miRNAs as early diagnostics biomarkers in CAD^12,13^.

Our previous work showed that microRNAs miR-128-3p & miR-195-5p regulate lipid metabolism. MiR-195 has been shown to regulate cellular cholesterol and triglyceride levels by targeting ACACA, FASN, HMGCR and CYP27B1 the key genes involved in fatty acid and cholesterol synthesis^14^. MiR-128-3p regulates cholesterol homeostasis by directly targeting Cholesterol efflux via binding to 3′ UTR (untranslated region) of ATP-binding cassette transporters (ABCA1 and ABCG1)^15,16^. Since lipid metabolism and transport play a major role in development and progression of coronary artery disease, we hypothesize that miR-128-3p & miR-195-5p might be ideal candidates for biomarkers for CAD.

The clinical diagnostic potential of these circulatory microRNAs has not yet been evaluated. Therefore, the present study was designed to explore the diagnostic biomarker potential of miR-128-3p and miR-195-5p for the first time in Indian cohort. In this study, we used serum of CAD patients as well as healthy control subjects and checked for the expression of miR-195-5p and miR-128-3p using quantitative Real Time-PCR (qRT-PCR). Further, using bioinformatics analysis and current literature we show here the important role of these micro-RNAs in CAD. 

## Material and methods

### Study population

A total of 124 cases, 89 CAD patients (69 male and 20 female) and 35 control individuals (12 male and 23 female) aged 30–76 years were recruited for the study from Department of Cardiology, AIIMS, New Delhi between March 2022 to February 2024. Eighty-nine patients were clinically suspected and angiographically proven cases of CAD with stenosis > 50% and followed the inclusive criteria. Patients with dilated cardiomyopathy, chronic kidney disease, other valves and genetic malformations of the heart were excluded from the study. Control participants of similar age group were angiographically shown to have < 10% stenosis. Clinicopathological history of all the subjects and their medication records were procured from the Department of Cardiology, AIIMS.

### Ethics statement

The protocols adopted were according to the ethical principles of the declaration of Helsinki. The study protocol complies with all the relevant national regulations (Indian Council of Medical Research, ICMR, India), Institutional Ethical Committee of CSIR-IGIB, Delhi and AIIMS, New Delhi (IEC-153/06.03.2020, RP-33/2020). A written and signed informed consent form was obtained from all the participants.

### Sample collection and serum isolation

5 ml of peripheral blood was collected from the subjects in primary blood collection tube with clot activator (S-Monovette® Serum-Gel 5 ml tubes, Sarstedt, Germany, cat. no. 02.1388). Serum was separated using two rounds of centrifugation (1900×*g* at 4 °C for 10 min and then 16,000×*g* at 4 °C for 10 min). The serum was collected and stored at − 80 °C until further processing.

### Biochemical profiling

Biochemical parameters including serum HbA1c (%), Total Cholesterol (TC), (mg/dl), Triglyceride (TG), (mg/dl), High density lipoproteins (HDL-C) (mg/dl), Low density lipoproteins (LDL-C) (mg/dl), Very low-density lipoproteins (VLDL-C) (mg/dl) were analyzed in the SMART Lab at AIIMS, New Delhi on automated chemistry analyzers.

### RNA isolation, cDNA synthesis and quantitative real time PCR

RNA was isolated from serum using miRNeasy serum/plasma advanced kit (Qiagen, USA, Cat No. 217204) as per manufacturers protocol. cel-miR-39 (Norgen Biotek, Canada, Cat No. 59000) was used as a spike-in control. cDNA preparation was done using High-Capacity cDNA Reverse Transcription Kit (Applied Biosystems, USA, Cat No. 4368814) as per manufacturer’s protocol. Stemloop RT primers were used for cel-miR-39, miR-128-3p and miR-195-5p. Primer sequences are mentioned in Table 1.

**Table 1:** List of Primers and sequences.

miRNA	Stem loop RT primer (5′ to 3′)	Forward primer (5′ to 3′)	Reverse primer (5′ to 3′)
cel-miR-39	CTCAACTGGTGTCGTGGAGTCGGCAATTCAGTTGAGCAAGCTGA	GAGCAGTCACCGGGTGTAAATCAG	GTGTCGTGGAGTCGGCAATTC
hsa-miR-128-3p	CTCAACTGGTGTCGTGGAGTCGGCAATTCAGTTGAGAAAGAGAC	GGAGTGTCACTTGGCCAGAG	GTGTCGTGGAGTCGGCAATTC
hsa-miR-195-5p	CTCAACTGGTGTCGTGGAGTCGGCAATTCAGTTGAGGCCAATAT	ACACTCCAGCTGGGTAGCAGCACAGAAAT	GTGTCGTGGAGTCGGCAATTC

For qRTPCR, reaction mixture was prepared using Kapa SYBR fast (Sigma-Aldrich, USA, Cat no. KM4101) dye as per manufacturer’s protocol and PCR was done on Roche lightcycler96 (Roche Diagnostics, Switzerland).

### Bioinformatic analysis

The list of gene targets for microRNA hsa-miR-128-3p were downloaded from databases such as targetscan 8.0, miRDB 6.0 and miRTarBase 9.0. A list of common gene targets were obtained from a Venn-diagram using Venny 2.1 (https://bioinfogp.cnb.csic.es/tools/venny). The common targets were further uploaded to ShinyGO database (http://bioinformatics.sdstate.edu/go/) for pathway analysis. Top KEGG pathways and top GO (Gene Ontology) Biological Processes were selected for further biological experiments^17–19^.

### Cell culture

THP-1 macrophage cells were cultured using RPMI 1640 medium (Ref no. R4130, Thermo Fisher Scientific, MA, USA) supplemented with 10% Fetal Bovine Serum (Ref no. 10082-147, Thermo Fisher Scientific, MA, USA) and 100 IU/mL penicillin–streptomycin (Gibco, Thermo Fisher Scientific, MA, USA). The cells were incubated at 37 °C and 5% CO_2_ level. For differentiation into macrophages, the cells were treated with 100 nm PMA (phorbol 12-myristate 13-acetate) for 48 h. Transient transfection was carried out in differentiated THP-1 cells for further experiments. Cells were transfected with miR-128 plasmid^20^ or antimiR-128-3p (AM-128) (AM17000, assay ID AM11746, Thermo Fisher Scientific, MA, USA) using Ingenio Electroporation Kit (MIR 50112, mirus bio, USA) as per manufacturer’s instructions. Cells transfected with pSilencer 4.1 vector and anti-miR negative control (NC) was used as control for miR-128-3p plasmid and antimiR-128-3p respectively^15^.

### Cholesterol efflux assay

The cholesterol efflux of differentiated THP-1 macrophages was examined using cholesterol efflux assay kit (ab196985, Abcam, USA) following the manufacture's instruction. Briefly, transfected THP-1 cells post 24 h were labelled with cholesterol for 1 h. After labelling cells were kept in equilibration buffer for 24 h and were then loaded with cholesterol acceptors for 4 h. Finally, cells were lysed and both the supernatant and lysate were transferred to a 96 well plate for measuring fluorescence (Ex/Em = 485/523 nm).

### Statistical analysis

Descriptive data on the participants recruited was summarized using measures of central tendency and dispersion. The normality of data was analysed using the Shapiro–Wilk test which was significant (non-parametric) in the case of CAD patients. Therefore, the differences between patient and control groups were tested using the Mann–Whitney *U* test. The expression levels of the miRNAs were calculated using the delta CT method. All the graphs were constructed using GraphPad prism version 8.1.1 (GraphPad software, San Diego, CA, USA). The Receiver Operating Characteristic (ROC) curve analysis was done and the respective area under curve (AUC) was calculated in order to assess the diagnostic potential of the circulatory miRNAs between CAD patients and healthy control group. Multiple logistic regression was done to adjust the effect of potential confounders of the study on miRNA expression. Pearson correlation analysis was done for correlation of miRNA expression and other cardiovascular risk factors associated with the subjects involved in the study. All statistical tests were done using GraphPad prism version 8.1.1 (GraphPad software, San Diego, CA, USA).

## Results

### Clinical characteristics along with CAD risk factors among the study participants.

A total of 124 cases (89 CAD patients and 35 control individuals) were recruited for the study. Age of participants showed an average of 55.90 ± 9.92 (CAD group) and 50.32 ± 8.25 (Control group). Gender, body mass index (BMI), Diabetes mellitus status (DM), hypertension, blood lipid profiles were recorded (Table 2). The male gender was more represented in CAD groups with 69 (77.53%) as compared to 12 (34.28%) in the control group, respectively. Total cholesterol, LDL, HDL, VLDL, Triglyceride, other medications and therapies were also recorded as shown in Table 2. 95% of CAD patients were on Aspirin, 92.5% on Statin and 90% were on beta blockers whereas 63.4% were taking ACE inhibitors and 14.6% were on Angiotensin receptor blockers. On the other hand, 53.8% of Control subjects were on Statin, 46% on beta blockers, 76.9% were taking Aspirin, 30.8% were taking Angiotensin receptor blockers and only 15.4% were taking ACE inhibitors.

**Table 2:** Baseline clinical characteristics of all the study subjects.

Parameters	CAD (n = 89)	Controls (n = 35)	p-value
Age	55.90 ± 9.92	50.32 ± 8.25	0.002
Gender (male/female)	69/20	12/23	< 0.0001
Height (in cm)	162.59 ± 7.03	158.60 ± 9.35	0.143
Weight (in kg)	67.35 ± 12.65	64.75 ± 10.31	0.750
BMI	25.27 ± 4.34	25.84 ± 4.14	0.359
Hypertension	61.5%	41.7%	0.148
Type 2 DM presence	46%	30.4%	0.225
Blood lipid profile			
Total cholesterol (mg/dl)	130.7 ± 44.16	174.27 ± 58.59	< 0.005
Triglyceride (mg/dl)	134.15 ± 34.96	141.14 ± 99.79	0.079
LDL (mg/dl)	62.9 ± 35.1	103.62 ± 62.67	0.032
HDL (mg/dl)	34.73 ± 8.44	44.8 ± 7.99	0.0002
VLDL (mg/dl)	25.22 ± 7.73	28.71 ± 19.62	0.361
Medications & therapies			
Aspirin	95%	76.9%	0.088
Statin	92.5%	53.8%	0.004
Beta blockers	90%	46.1%	0.009
Ace inhibitors	63.4%	15.4%	0.023
Angiotensin receptor blockers	14.6%	30.8%	0.237

*Data has been represented in the form of percentages and Mean ± SD. The statistical significance of the clinical parameters was analysed using non-parametric Mann–Whitney *U* test. Age, Gender, Total cholesterol, LDL, HDL, Statin, Beta blockers and Ace Inhibitors were significantly different between control subjects and the patients.

### Circulating miR-128-3p and miR-195-5p levels in CAD vs. control subjects

**Figure 1:**
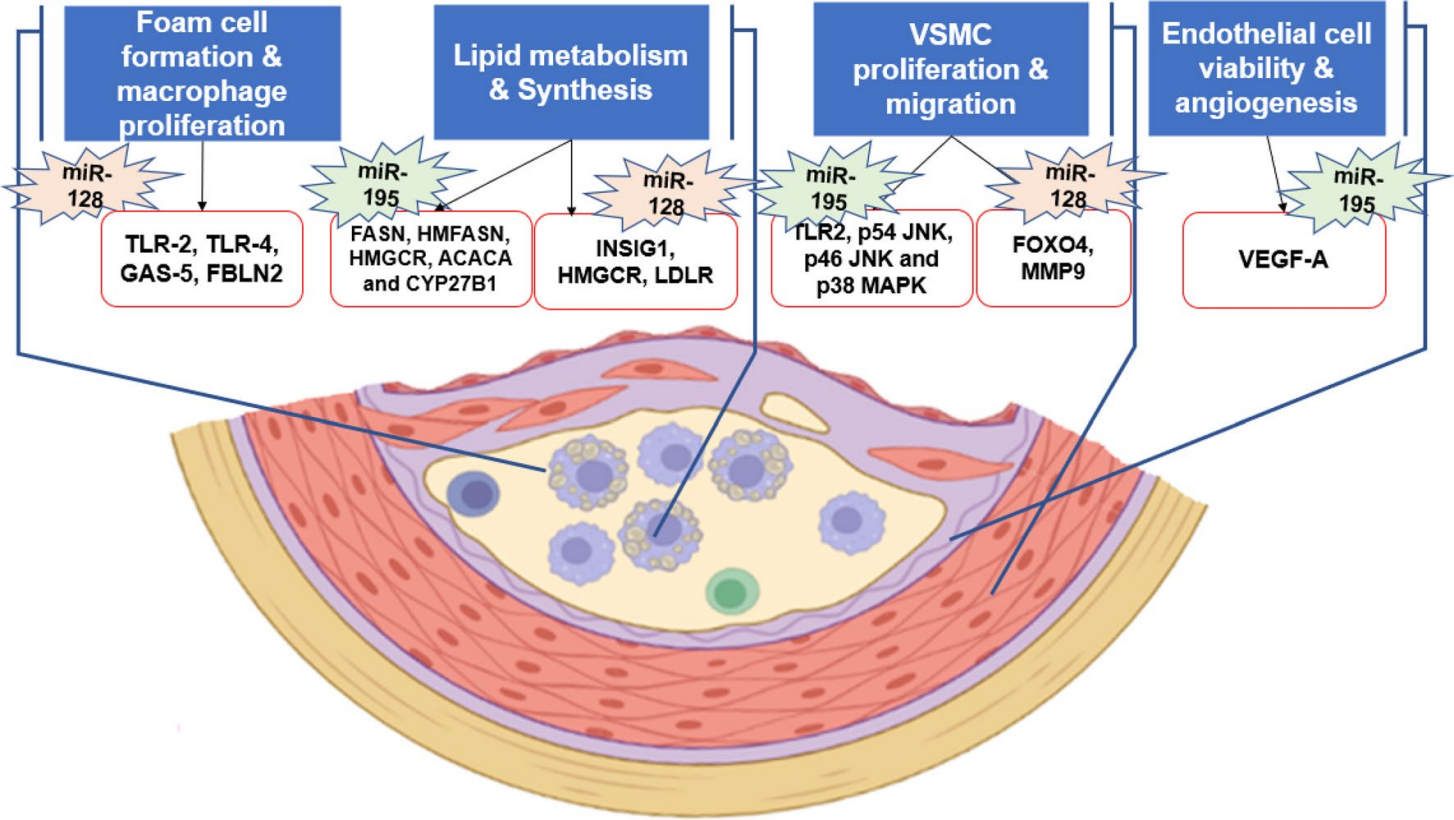
Functionally validated target genes of miR-128-3p and miR-195-5p in coronary artery disease. The figure is a representation of the role of both miR-195 and miR-128 in various aspects of coronary artery disease. The validated target genes of each miRNA with respect to atherosclerosis and other cardiovascular disorders are shown.

We first did an extensive review of literature for finding if there are any validated targets of miR-128-3p and miR-195-5p that might play a role in various aspects of coronary artery disease^14–16,21–23^. As shown in Fig. 1, both miR-128 and miR-195 target genes affecting major cell types involved in CAD i.e., macrophages, vascular smooth muscle cells and endothelial cells. Both miRNAs also target genes involved in lipid metabolism and synthesis as well as vascular smooth muscle proliferation and migration. Hence, we further evaluated the expression level of both miR-128-3p and miR-195-5p in patients with established CAD and control subjects using quantitative Real Time-Polymerase Chain Reaction (qRT‐PCR).

**Figure 2:**
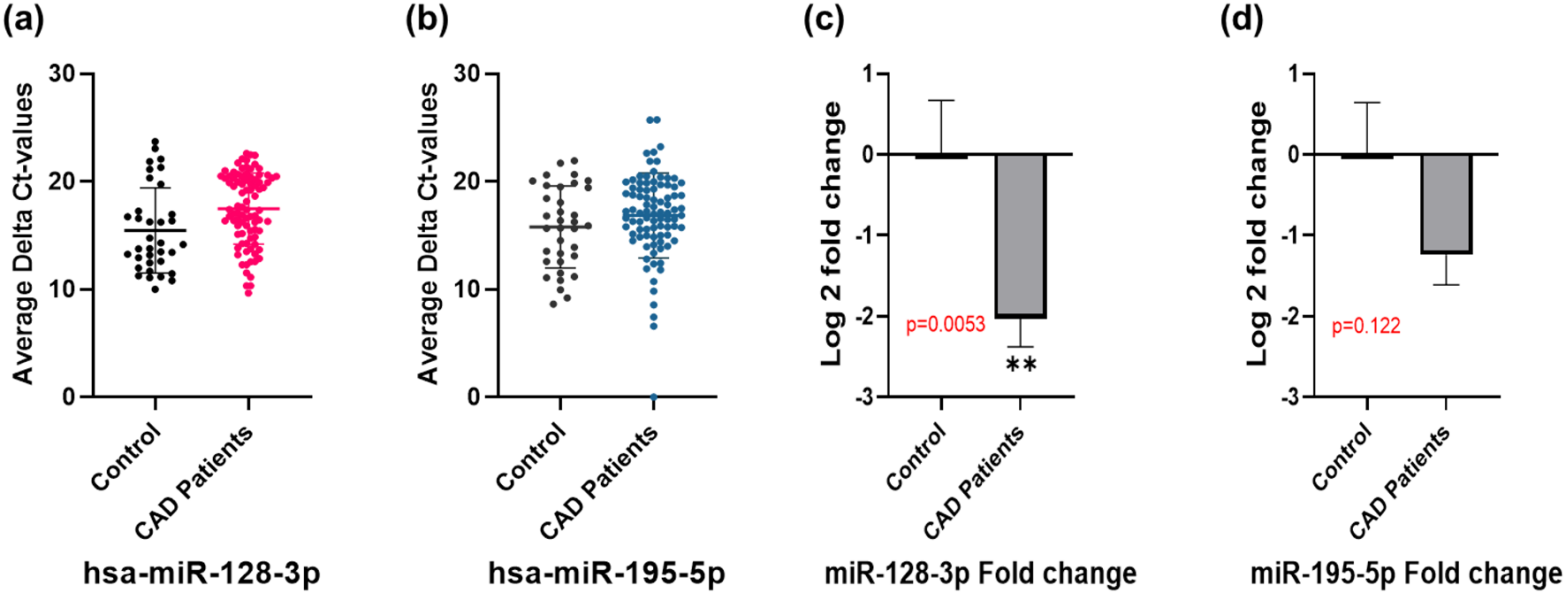
Differential Expression pattern of miR-128-3p & miR-195-5p: (**a**) Expression levels of miR-128-3p in CAD patients (n = 89) in comparison to control subjects (n = 35) in terms of average delta Ct values. (**b**) Expression levels of miR-195-5p in CAD patients (n = 89) vs. control subjects (n = 35) in terms of average delta Ct values. Results were measured as Mean ± SD. (**c**) Relative Fold change of miR-128-3p in CAD patients as opposed to control subjects. It was significantly decreased with a p value of 0.0053. Results are (Mean ± SEM) (**d**) Relative fold change of miR-195-5p in CAD patients when compared to control subjects. miR-195-5p levels did not show any significant changes (p value = 0.122) Results are (Mean ± SEM).

As shown in Fig. 2a, we found the average delta-Ct values of miR-128-3p to be 17.50 ± 3.28 in CAD patients in contrast to 15.47 ± 3.89 in control subjects. In case of miR-195-5p the average delta-Ct values ranged from 16.85 ± 3.92 in case of CAD patients and 15.79 ± 3.74 in case of control subjects as depicted in Fig. 2b. Relative expression pattern [log2 fold change (Mean ± SEM)] of both the microRNAs have been depicted in Fig. 2c and d. Comparing the expression levels of both the miRNAs using Mann–Whitney *U* test, we can see that miR-128-3p is 2.03 ± 0.35 fold (p-value = 0.0053) decreased in CAD patients as compared to control subjects, whereas miR-195-5p was shown to be 1.24 ± 0.37 fold (p value = 0.1221) decreased in CAD patients vs control. Therefore, our findings were significant for miR-128-3p, but not in the case of miR-195-5p.

### Assessment of circulating miR-128-3p and miR-195-5p for their diagnostic potential

**Figure 3:**
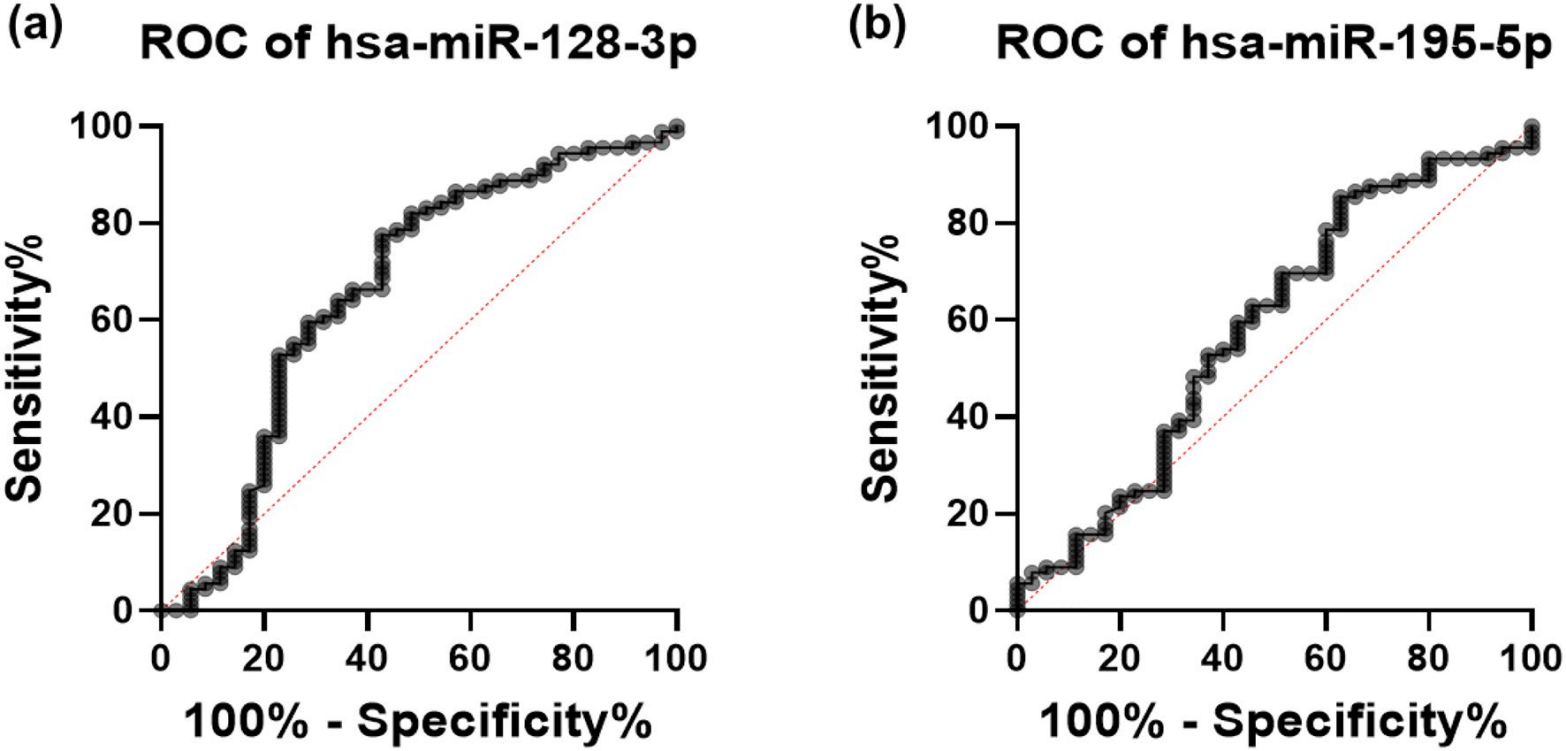
Receiver operating Characteristic (ROC) curve: diagnostic potential of miR-128-3p and miR-195-5p in coronary artery disease depicted by Receiver operating characteristic curve. (**a**) Area under Curve (AUC) of miR-128-3p is 0.6602, 95% confidence interval is 0.5421 to 0.7782 while the p value is 0.0056. (**b**) ROC curve of miR-195-5p with an AUC of 0.5841, 95% confidence interval is 0.4668 to 0.7014 and p Value is 0.1458.

We conducted receiver-operating-characteristic (ROC) curve analysis to further explore the potential and application of these circulatory miRNAs (miR-128-3p and miR-195-5p) as novel and possible diagnostic biomarkers of CAD (Fig. 3). A reasonably high predictive value/area under the curve (AUC) was obtained in CAD patients in the case of miR-128-3p.

The area under the curve of miR-128-3p was 0.6602 with a standard error of 0.06023 (p value 0.0056) and 95% confidence interval was between 0.5421 to 0.7782 (Fig. 3a). This implies that miR-128-3p has a reasonable predictive value (66%) for differentiating between CAD patients and healthy controls. In the case of miR-195-5p the area under the curve was 0.5841 with a standard error of 0.05986 (p value 0.1458, non-significant) and 95% confidence interval was between 0.4668 to 0.7014 (Fig. 3b). The optimal cut-off point for each miRNA was determined using the largest sum of sensitivity and specificity which was 14.81 for miR-128-3p and 13.94 for miR-195-5p^24^. The detail of this analysis for both miRNAs is provided in Table 3.

**Table 3:** Receiver operating characteristics (ROC) curve analysis of miR-128-3p and miR-195-5p for their potential as a diagnostic marker for CAD.

	AUC (95% CI)	Cut-off value	Likelihood ratio	Sensitivity (95% CI)	Specificity (95% CI)
miR-128-3p	0.6602 (0.5421–0.7782)	14.81	1.809	77.53% (67.82–84.96)	57.14% (40.86–72.02)
miR-195-5p	0.5841 (0.4668–0.7014)	13.94	1.359	85.39% (76.60–91.26)	37.14% (23.17–53.66)

As a result, our findings imply that serum levels of miR-128-3p may be useful as a prospective diagnostic biomarker for the identification and prediction of CAD patients.

### Multivariate analysis of miR-128-3p

To study the interrelationship between miRNA expression and classical CAD risk factors we did Pearson’s correlation analysis of these factors with miR-128-3p delta ct- values. No significant correlation was found between miR-128 and gender, BMI, hypertension, Diabetes Mellitus, LDL, VLDL, Triglycerides, Total Cholesterol. Poor correlation was found for age (R squared 0.06, p-value 0.005) and HDL (R squared 0.07, p-value 0.04).

**Figure 4:**
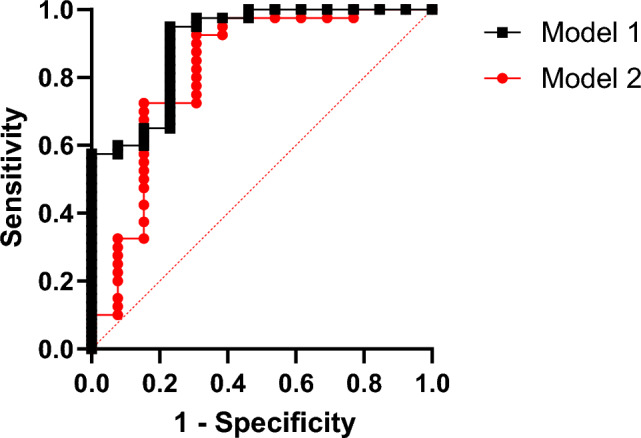
Multivariate Analysis: Comparison between ROCs of two models used for multivariate analysis. Model 1 (AUC 0.9019) has miR-128-3p and other confounding factors such as age, gender, statin, aspirin, ARB, beta blocker, ACE inhibitors. Model 2 (AUC 0.8212) contains all the confounding factors of Model 1 except miR-128. Model 1 shows a better predictive value than model 2.

MiR-128-3p was further evaluated as a potential independent predictor of coronary artery disease (CAD) using multiple logistic regression. The model adjusted for confounding factors, including the use of aspirin, statins, Beta-blockers, Ace-Inhibitors and angiotensin receptor blockers (ARBs), which are commonly prescribed in the patient cohort. MiR-128-3p demonstrated a statistically significant association with the presence of CAD (Log-likelihood ratio (G squared) test, p-value 0.034), suggesting its potential role as an independent biomarker. The odds ratio (OR 1.241, 95% CI 0.8566 to 1.901) adjusted for the confounding medications indicated that an increase in miR-128 delta-ct values was correlated with an elevated risk of CAD, independent of the drug regimen. Figure 4 presents a comparative analysis of the Receiver Operating Characteristic (ROC) curves for two multivariate regression models. The inclusion of miR-128-3p alongside other potential confounders in the model1 results in a superior predictive performance, as indicated by a higher Area Under the Curve (AUC) value of 0.9019, compared to the model 2 that accounts only for the confounding factors, which has an AUC of 0.8212.

### Function and pathway enrichment analysis of miR-128-3p

**Figure 5:**
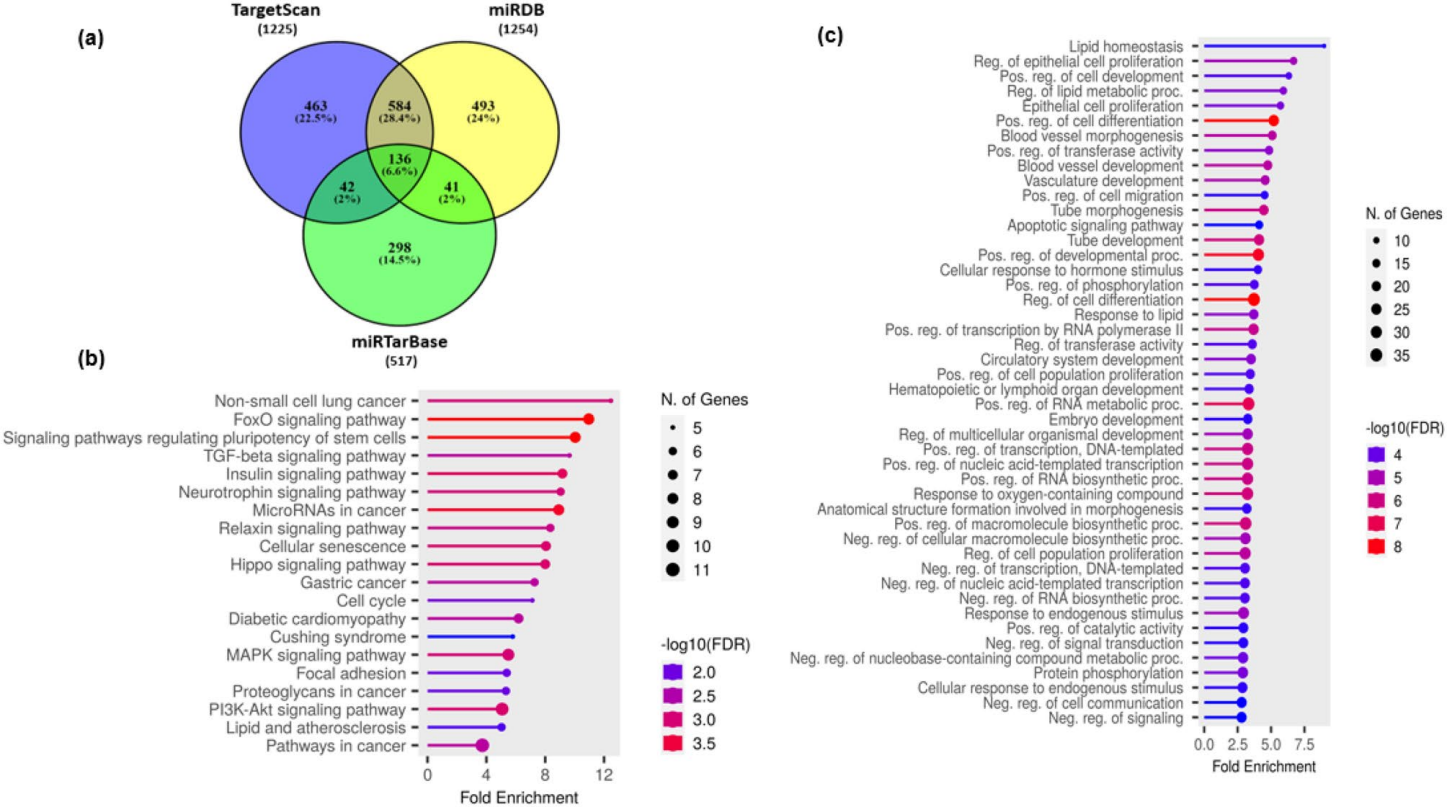
Bioinformatic analysis of predicted targets of miR-128-3p: (**a**) Venny tool was used to create a Venn diagram of common targets of miR-128-3p from three different target prediction tools TargetScan8.0, miRDB6.0, miRTarBase. (**b**) KEGG pathway analysis of the overlapping genes done using ShinyGO 0.77 shows Lipid and Atherosclerosis as a significant pathway with an FDR of 0.013. (**c**) Gene Ontology (GO)enrichment analysis of top 50 Biological Processes of common targets was also done using ShinyGO 0.77.

Using target prediction tools such as TargetScan, miRDB, miRTarBase we explored the top pathways regulated by miR-128-3p. Comparing different databases we found a total of 136 common predicted targets of miR-128-3p (Fig. 5a, Supplementary Table 1). The common targets were further uploaded to ShinyGO database (http://bioinformatics.sdstate.edu/go/) for pathway analysis. Figure 5b shows a list of the top 20 KEGG pathways as mentioned in Table 4. Several pathways directly related to CAD such as Insulin signaling pathway, diabetic cardiomyopathy pathway, lipid and atherosclerosis pathway were observed to be regulated by miR-128-3p. In order to further understand the role of these genes in the pathophysiology of CAD we also performed GO Enrichment analysis for top 50 biological processes (Fig. 5c). The common target genes were highly enriched in lipid homeostasis. The target genes of these pathways have been mentioned in Table 5. Some of the proteins such as LDLR, ABCA1, RXRA have been previously shown to be the direct target of miR-128-3p by our lab^15^ (Table 5).

**Table 4:** Top 20 KEGG pathways sorted by their fold enrichment of miR-128-3p obtained from common gene targets.

Pathway	Enrichment FDR	Number of genes	Pathway genes	Fold enrichment
Non-small cell lung cancer	0.001069	5	72	12.47
FoxO signaling pathway	0.000137	8	131	10.96
Signaling pathways regulating pluripotency of stem cells	0.000137	8	143	10.04
TGF-beta signaling pathway	0.002455	5	93	9.65
Insulin signaling pathway	0.000578	7	137	9.17
Neurotrophin signaling pathway	0.001069	6	119	9.05
MicroRNAs in cancer	0.000221	8	161	8.92
Relaxin signaling pathway	0.001522	6	129	8.35
Cellular senescence	0.000834	7	156	8.05
Hippo signaling pathway	0.000834	7	157	8
Gastric cancer	0.002455	6	148	7.28
Cell cycle	0.008574	5	126	7.12
Diabetic cardiomyopathy	0.002347	7	203	6.19
Cushing syndrome	0.017264	5	155	5.79
MAPK signaling pathway	0.000994	9	294	5.49
Focal adhesion	0.010299	6	200	5.38
Proteoglycans in cancer	0.010299	6	202	5.33
PI3K-Akt signaling pathway	0.000834	10	354	5.07
Lipid and atherosclerosis	0.013125	6	214	5.03
Pathways in cancer	0.002455	11	530	3.73

**Table 5:** Gene lists of top 20 KEGG pathways of miR-128-3p obtained from the common gene targets.

Pathway	Genes
Non-small cell lung cancer	E2F3 SOS1 PDPK1 RET RXRA
FoxO signaling pathway	SIRT1 TGFBR1 MAPK14 SOS1 PDPK1 G6PC3 SETD7 IRS1
Signaling pathways regulating pluripotency of stem cells	ISL1 MAPK14 SMAD5 ZFHX3 WNT3A BMI1 SMAD2 BMPR2
TGF-beta signaling pathway	TGFBR1 SMAD5 SMAD2 SP1 BMPR2
Insulin signaling pathway	MKNK2 SOS1 PDPK1 G6PC3 PDE3B IRS1 PPP1CC
Neurotrophin signaling pathway	RPS6KA5 MAPK14 SOS1 RAP1B PDPK1 IRS1
MicroRNAs in cancer	SIRT1 RPS6KA5 E2F3 SOS1 WNT3A BMI1 IRS1 BMPR2
Relaxin signaling pathway	TGFBR1 MAPK14 SOS1 VEGFC GNG12 SMAD2
Cellular senescence	SIRT1 TGFBR1 MAPK14 E2F3 SMAD2 ZFP36L1 PPP1CC
Hippo signaling pathway	TGFBR1 WNT3A YWHAB MOB1B SMAD2 PPP1CC BMPR2
Gastric cancer	TGFBR1 E2F3 SOS1 WNT3A SMAD2 RXRA
Cell cycle	E2F3 STAG1 WEE1 YWHAB SMAD2
Diabetic cardiomyopathy	TGFBR1 PPIF MAPK14 IRS1 SMAD2 SP1 PPP1CC
Cushing syndrome	E2F3 RAP1B LDLR WNT3A SP1
MAPK signaling pathway	MKNK2 RPS6KA5 TGFBR1 MAPK14 SOS1 RAP1B VEGFC GNG12 CSF1
Focal adhesion	SOS1 RAP1B PDPK1 VEGFC PPP1CC RELN
Proteoglycans in cancer	MAPK14 SOS1 PDPK1 WNT3A SMAD2 PPP1CC
PI3K-Akt signaling pathway	SOS1 PDPK1 G6PC3 VEGFC YWHAB IRS1 GNG12 CSF1 RXRA RELN
Lipid and atherosclerosis	MAPK14 RAP1B LDLR PDPK1 ABCA1 RXRA
Pathways in cancer	RPS6KA5 TGFBR1 E2F3 SOS1 VEGFC WNT3A RET GNG12 SMAD2 SP1 RXRA

In our study miR-128-3p has been shown to be significantly decreased in CAD patients as compared to healthy controls. Our current findings strongly suggest that miR-128-3p could be a potential biomarker for CAD diagnosis. However, the sensitivity and specificity of our current circulating miR-128-3p need to be further investigated in a larger cohort.

### MiR-128 is involved in the regulation of cholesterol efflux from macrophages

**Figure 6:**
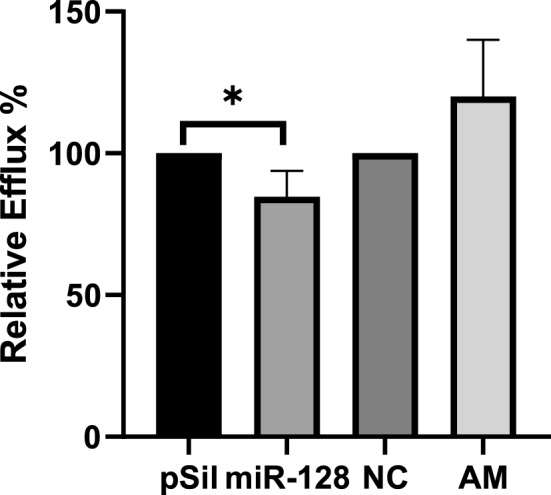
Regulation of Cholesterol Efflux in macrophage cells by miR-128-3p: Figure represents cholesterol efflux % of differentiated THP-1 macrophage cells in during overexpression of miR-128-3p (miR-128) and Inhibition using anti-miR-128-3p (AM). Overexpression of miR-128-3p significantly decreases cholesterol efflux by 15.27% as compared to pSil (pSilencer4.1) control. Inhibition of miR-128-3p increased the efflux by 20.10% in comparison to the negative control.

It is evident from the bioinformatic analysis that miR-128 plays an important role in the regulation of cholesterol homeostasis by targeting key genes involved in cholesterol transport. Cholesterol homeostasis regulates the rate of lipid uptake and accumulation by macrophages to form foam cells which is the preliminary major event in the development of coronary artery disease^1^. Reverse Cholesterol Transport (RCT) or cholesterol efflux in macrophages attenuates the formation of foam cells and thus reverses the condition of CAD^1^. Therefore, we decided to investigate the role of miR-128 in modulating cholesterol efflux in macrophage cells (THP-1 cells) in vitro (n = 3). From the results we can see that overexpression of miR-128 significantly reduces the cholesterol efflux by 15.27 percent as compared to control (pSilencer) (p value 0.04). Inhibition of miR-128 using antimiR increased the efflux by 20.10% as compared to negative control (NC) (p value 0.15) (Fig. 6).

## Discussion

Due to their small size and protection from endogenous RNase activity provided by their association with certain lipid-based carriers or their encapsulation within micro vesicles, circulating miRNAs are extremely resistant to destruction^25^. Circulatory miRNAs have become a major and intriguing prospect for the discovery of novel regulators of cardiovascular illnesses, including CAD, thanks to their benefits over other traditional methods of diagnostics^25,26^.

This pilot study based on the Indian cohort was aimed at identification of novel microRNA-based biomarkers in coronary artery disease. To summarize our findings, miR-128-3p was found to be significantly decreased in CAD patients as compared to control whereas no significant difference was observed in case of miR-195-5p. The diagnostic potential evaluated using the ROC curve has shown miR-128-3p to have 66% accuracy (AUC 0.6602) in distinguishing CAD patients from control.

Several micro RNAs have been reported over the years which regulate coronary artery disease^27–32^. Some of these micro RNAs are present in circulation and few have been reported to be differentially expressed in serum/plasma^30–33^. To report a few, Ling et al. found that the exosomal miR-126 levels were positively correlated in cases of unstable angina (n = 31) as well as acute myocardial infarction (n = 34)^34^. In another study by O′Sullivan et al. on a sample set of 150 patients, miR-93-5p was reported to be significantly upregulated in stable CAD when compared to the control group^35^. However, most of these studies have been carried out in Caucasian population. In this study we have reported two miRNAs miR-128-3p and miR-195-5p in Indian population. These miRNAs have a known role in metabolic disorders as described previously. As per our results only miR-128-3p can clearly differentiate between CAD and control population. MiR-128-3p serum levels has shown negative correlation with respect to risk of CAD. Similar to our findings, few microRNAs have also been reported in Indian population such as Reddy et al. in 2019 reported miR-33 to be overexpressed by 2.9 folds in CAD patients than in control group (n = 60)^36^. Kumar et al. in 2020 reported that miR-133b & miR-21 as a predictive biomarker for coronary artery disease (AUC 0.8 & 0.79 respectively) in a cohort of 147 subjects^37^.

To further elucidate the role of miR-128-3p in coronary artery disease we investigated its potential gene targets and major affected signaling pathways using several bioinformatic tools. Our results showed that miR-128-3p was significantly associated with lipid homeostasis. In silico analysis using publicly available databases identified several genes related to cholesterol metabolism and efflux that were inversely correlated with miR-128-3p expression. These results indicate that miR-128-3p may have a broader impact on cholesterol homeostasis.

Cholesterol efflux is a key process for maintaining cellular cholesterol homeostasis and preventing atherosclerosis^38^. Perturbation of homeostasis leads to increased accumulation of cholesterol resulting in foam cell development and exacerbation of a pro-inflammatory state that in turn promotes atherosclerosis development. Cholesterol efflux is mediated by ATP-binding cassette transporters (ABCA1 and ABCG1) that transfer cholesterol to apolipoprotein A-I (apoA-I) and high-density lipoprotein (HDL) particles, respectively^39^. The expression of ABCA1 and ABCG1 is regulated by nuclear receptors such as liver X receptor (LXR) and retinoid X receptor (RXR)^39^. Previous studies have shown that microRNAs (miRNAs) can modulate the expression of genes involved in cholesterol metabolism and efflux^27,40^. In a study done by Rayner et al., miR-33 was shown to inhibit the expression of ABCA1 and ABCG1, thereby repressing cholesterol efflux to ApoA1 and HDL, respectively^41^. Jin et al. showed that miR-19a-3p regulates apoptosis and cholesterol efflux through down-regulation of SDC-1, ABCA1, ABCG1, TGF-β1 and p-Smad3 proteins in ox-LDL-induced HAECs^42^.

In our previous lab findings, it was reported that miR-128-3p directly targets ABCA1, ABCG1 and RXRα, and reduces their expression levels in different cell types^15^. We hypothesized that miR-128-3p may modulate cholesterol efflux and affect the development of atherosclerosis in CAD patients. To validate the functional role of miR-128-3p in cholesterol efflux, we performed an efflux assay using THP-1 macrophages transfected with either miR-128 plasmid or inhibitor. We found that overexpression of miR-128 significantly decreased the cholesterol efflux to cyclodextrin, whereas inhibition of miR-128 increased the efflux. Together our findings confirmed that miR-128 negatively regulates cholesterol efflux in macrophages.

In this study, we found that overexpression of miR-128 inhibited the expression of genes involved in regulation of cellular cholesterol homeostasis in macrophages. However, we also observed that miR-128-3p was decreased in the extracellular secretion in serum of patients with CAD. These contrasting results may be due to miR-128’s varied regulatory effects across distinct cell types implicated in CAD. Previous studies have shown that miR-128-3p can modulate the function of endothelial cells and Vascular smooth muscle cells (VSMCs) which are also involved in CAD development and progression^43^. For instance, miR-128-3p can inhibit endothelial cell proliferation and migration by targeting VEGF1 and can regulate proliferation, differentiation, and migration in VSMCs^43,44^. Therefore, the net effect of miR-128-3p on CAD may depend on the balance between its actions on different cell types.

### Study limitations

Our study highlights promising data on the dysregulation of miR-128-3p in CAD and its potential role in cholesterol regulation. However, there are several limitations to consider. The research was based on a small group of CAD patients and healthy individuals, necessitating further validation of hsa-miR-128-3p’s diagnostic accuracy in a broader sample. Moreover, the lack of longitudinal data calls for additional studies to explore the prognostic implications of hsa-miR-128-3p. To ensure the findings are generalizable, it’s crucial to replicate the study across diverse cohorts in various regions of India.

CAD is a complex disease influenced by genetic and environmental factors. Understanding miR-128-3p’s role in CAD is crucial for validating its impact on the disease’s progression. The effects of epigenetic changes, especially those caused by related conditions like hypertension and diabetes or medications, are not fully understood^44^. Additionally, CAD’s complexity, involving interactions among proteins in different cellular pathways, underscores the need to confirm our findings across various cell types (VSMCs, endothelial cells) involved in CAD development and pathogenesis.

## Conclusion

In our study miR-128-3p has been shown to be decreased in CAD patients as compared to healthy individuals. Our study, which included 124 subjects, provides evidence that miR-128-3p is significantly correlated to the chance of developing CAD. We have shown miR-128-3p to regulate cholesterol efflux in macrophages which is an important event in the development of coronary artery disease. Examination of exosomal miR-128-3p and validation of the status of miR-128-3p in patient cohorts stratified by severity of atherosclerosis could be explored for better understanding in patients with CAD in further studies.

### Supplementary Information

Supplementary Table 1.

## Data Availability

The qRT-PCR data generated in the current study is available from the corresponding author on reasonable request.

## Abbreviations

CAD: Coronary artery disease
ECG: Electrocardiogram
cTnT: Cardiac troponin
CK-MB: Creatine kinase
CRP: C-reactive protein
ROC: Receiver’s operating characteristic
AUC: Area under curve
TC: Total cholesterol
TG: Triglyceride
HDL: High density lipoproteins
LDL: Low density lipoproteins
VLDL: Very low-density lipoproteins
