# Supplementary material for: Role of miR-128-3p and miR-195-5p as biomarkers of coronary artery disease in Indians: a pilot study
Source: Sci Rep. 2024 May 24;14:11881. doi: 10.1038/s41598-024-61077-4 (PMC11126699; doi:10.1038/s41598-024-61077-4)
Supplement: Supplementary file 1 — Supplementary Table 1. [file 41598_2024_61077_MOESM1_ESM.docx]

**Supplementary Table 1**

A list of 136 common gene targets of miR-128-3p identified using 3 target prediction tools, namely, TargetScan, miRDB and miRTarBase.

| **Common 136 gene targets of miR-128-3p** | | | |
| --- | --- | --- | --- |
| PAIP2 | AFF4 | GNG12 | CPEB3 |
| CLPP | RELN | PPIF | MPP2 |
| PDIA5 | PDHX | RET | PLAG1 |
| CASC3 | UBE2W | TGFBR1 | MBTD1 |
| YWHAB | ATP8A1 | KAT7 | CCNK |
| TMEM167A | SH3RF1 | DDX6 | SIRT1 |
| H3F3C | GPAM | LMNB1 | E2F3 |
| BAG2 | GCNT2 | WNT3A | SGPP1 |
| PITHD1 | FBXW7 | ADAMTS5 | FOXP4 |
| VEGFC | ZNF385A | ITSN2 | GRM5 |
| SFXN2 | MAPK14 | EIF5 | DCP2 |
| WEE1 | SLC39A11 | TUB | BMPR2 |
| H3F3B | CSRP2 | PDK1 | MKNK2 |
| MOB1B | NR2F2 | SMAD5 | RFX3 |
| SEC61A1 | LDLR | SERTAD2 | SLC7A11 |
| NEK2 | IRS1 | NEURL1B | KMT2C |
| UBE2N | FBLN5 | MIER3 | KPNB1 |
| AKIRIN1 | DCAF7 | USP46 | EIF2S2 |
| G6PC3 | NAA50 | STAG1 | TMEM170B |
| PPP1CC | NDST1 | ELL2 | INO80D |
| RNF182 | PDE3A | GFPT2 | SOS1 |
| ISL1 | UNC13C | SUCO | SLC5A3 |
| GATA6 | TNPO1 | PDPK1 | EPHB2 |
| ALDH4A1 | ZNF652 | HOXA10 | SUZ12 |
| RAP1B | CSF1 | SETD7 | NR1D2 |
| FAM84B | NUS1 | ZNF800 | ZFHX3 |
| SP1 | ARHGAP12 | LONRF1 | TMED5 |
| BMI1 | FUBP3 | DYNLL2 | PLAGL2 |
| RPS6KA5 | VPS4B | RREB1 | EPB41L1 |
| KBTBD11 | EYA4 | PDE3B | RXRA |
| ZFP36L1 | GOLM1 | HOXC6 | ZBTB20 |
| DCX | E2F7 | APPBP2 | TMBIM6 |
| UGCG | TMEM91 | ABCA1 | SMAD2 |
| GCC2 | STK35 | TXNIP | RGPD4 |
